# Mitochondrial p38β and Manganese Superoxide Dismutase Interaction Mediated by Estrogen in Cardiomyocytes

**DOI:** 10.1371/journal.pone.0085272

**Published:** 2014-01-22

**Authors:** Han Liu, Mounica Yanamandala, Tiffany C. Lee, Jin Kyung Kim

**Affiliations:** 1 Department of Medicine, University of California Irvine, Irvine, California, United States of America; 2 School of Medicine, University of California Irvine, Irvine, California, United States of America; Northwestern University, United States of America

## Abstract

**Aims:**

While etiology behind the observed acceleration of ischemic heart disease in postmenopausal women is poorly understood, collective scientific data suggest cardioprotective effects of the endogenous female sex hormone, estrogen. We have previously shown that 17β-estradiol (E2) protects cardiomyocytes exposed to hypoxia-reoxygenation (H/R) by inhibiting p38α - p53 signaling in apoptosis and activating pro-survival p38β mitogen activated protein kinase (p38β MAPK), leading to suppression of reactive oxygen species (ROS) post H/R. However, little is known about the mechanism behind the antioxidant actions of E2-dependent p38β. The aim of this study is to determine whether the cytoprotection by estrogen involves regulation of manganese superoxide dismutase (MnSOD), a major mitochondrial ROS scavenging enzyme, via cardiac p38β.

**Methods and Results:**

We identified mitochondrial p38β by immunocytochemistry and by immunoblotting in mitochondria isolated from neonatal cardiomyocytes of Sprague-Dawley rats. E2 facilitated the mitochondrial localization of the active form of the kinase, phosphorylated p38β (p-p38β). E2 also reduced the H/R-induced mitochondrial membrane potential decline, augmented the MnSOD activity and suppressed anion superoxide generation, while the dismutase protein expression remained unaltered. Co-immunoprecipitation studies showed physical association between MnSOD and p38β. p38β phosphorylated MnSOD in an E2-dependent manner in *in-vitro* kinase assays.

**Conclusion:**

This work demonstrates for the first time a mitochondrial pool of active p38β and E2-mediated phosphorylation of MnSOD by the kinase. The results shed light on the mechanism behind the cytoprotective actions of E2 in cardiomyocytes under oxidative stress.

## Introduction

Ischemic heart disease (IHD) is the leading cause of mortality worldwide [1], [2]. For reasons that are not completely understood, there exist pronounced gender differences in IHD. Compared to age-matched men, the risk of IHD in premenopausal women is much lower, only to equal or exceed that in men after menopause. This observation has led to a long-held belief that endogenous estrogen may be cardioprotective against the development and/or complications of IHD in women. In support of this notion, the predominant and most physiologically active form of estrogen, 17β-estradiol (E2), has been consistently shown to mitigate the extent of ischemia-induced injury in controlled experimental models [3].

One of the ways E2 protects the heart is by attenuating death of cardiomyocytes, differentiated heart muscle cells [4], [5]. It was previously shown that, in primary cultured murine cardiomyocytes subjected to simulated ischemia-reperfusion (I/R), E2 prevents cell death by activating p38β mitogen-activated protein kinase (p38β MAPK) via the phospho-inositide-3 kinase (PI3K)/Akt survival pathway [6]. The p38β MAPK in turn suppresses in an E2-dependent manner the production of reactive oxygen species (ROS), a potent trigger for apoptosis. When the endogenous p38β activity is suppressed via a dominant negative p38β mutant or use of a specific chemical inhibitor, E2 no longer inhibits ROS generated from hypoxia-reoxygenation (H/R) or protects the cell from undergoing apoptosis, underscoring a critical role p38β plays in the E2-mediated regulation of oxidative stress. In our recent work, we have also demonstrated a negative feedback mechanism between E2-activated p38β and proapoptotic p53 in mitochondria-driven apoptosis [7].

In the current study, we investigate the molecular mechanism behind the novel antioxidant properties of p38β responsible for the E2-mediated cardiomyocyte protection post hypoxic stress. The p38β MAPK, first characterized by Han and colleagues, shares 74% homology with p38α, the first described and best-known member of the p38MAPK family [8]. The functions of p38β, however, are distinct from those of p38α in many cellular contexts [6], [9], [10], [11], and encompass preservation of cytoarchitecture and participation in hypertrophic signaling. In contrast, p38α is often proapoptotic in its role. We first reported the antioxidant effects of p38β in cultured rat cardiomyocytes subjected to H/R, in which E2-mediated suppression of ROS and consequent myocyte protection was dependent on p38β [6]. However, how this kinase effects ROS inhibition is virtually unknown. While many p38β targets have been identified, with most being transcription factors or cytoskeletal proteins [12], little is known about a potential substrate for the kinase in the context of this antioxidative function. Much of ROS generation post H/R occurs at mitochondria, the subcellular site of energy production and redox homeostasis. Therefore, to investigate the mechanism behind the antioxidant property of p38β, we sought to delineate the presence and actions of this kinase in mitochondria.

We report here that there exists a mitochondrial pool of p38β in cardiomyocytes, and that the kinase phosphorylates a major mitochondrial antioxidant enzyme, manganese superoxide dismutase (MnSOD), in an estrogen-dependent manner. Through the use of specific agonists and inhibitors for each estrogen receptor (ER) subtype, we also show that the E2-mediated increase in the MnSOD activity is supported by both ERα and ERβ.

## Methods

### Harvest and culture of rat cardiomyocytes

The investigations conformed to the Guide for the Care and Use of Laboratory Animals published by the US National Institutes of Health (NIH Publication No. 85-23, revised 1996). All studies were approved by the Institutional Animal Care and Use Committee at the University of California, Irvine (Protocol # 2007-2755). All efforts were made to minimize suffering. One to two-day old rats (Sprague-Dawley) were anesthetized by isoflurane gas administered through a commercial rodent anesthetic machine (Summit Medical Equipment Company) in an induction chamber, with a mix of isoflurane and oxygen gas at the flow rate of 3 liter/min and 1 liter/min, respectively, under the hood. The level of anesthesia was assessed by response to toe pinch. Once adequate anesthesia was achieved, the animal was positioned on a procedure table and fitted through a nose cone adaptor that continued to deliver the anesthetic gas to the animal. Then, cardiomyocytes were isolated using a neonatal cardiomyocyte isolation system kit (Worthington), as reported previously [6], [7]. Briefly, anesthetized neonatal rats were prepped in sterile fashion and beating hearts quickly excised into a cold Hanks balanced salt solution before collagen-based stepwise enzymatic digestion per manufacturer's protocol. Once the heart was removed, the animal was considered euthanized (under anesthesia). Thus harvested cells were then incubated in DMEM/F12 (1∶1) cell culture medium (Gibco) supplemented with 10% fetal bovine serum (Invitrogen) and 1% antibiotic-antifungal (Sigma) at 37°C before use.

### Hypoxia-reoxygenation

Cells were synchronized in DMEM/F12 medium without the serum or the antibiotic-antifungal agent, pretreated for 30 minutes with indicated reagents, then subjected to H/R, as described previously [7]. Briefly, cells were placed overnight at 37°C in an air-tight hypoxia chamber (Billups-Rothenberg) flushed with 95% N2, 4% CO2, and 1% O2 to yield less than 2% oxygen, measured by an oxygen analyzer (Vascular Technology), followed by 2 hr reoxygenation by exposure to room air at 37°C. In experiments of hypoxia alone without a reoxygenation phase, cells were immediately placed on ice and processed for downstream assays after the aforementioned period of hypoxia without the reoxygenation period.

### Mitochondria isolation

Mitochondria from rat cardiomyocytes after indicated treatments were collected by using a Mitochondrial Isolation Kit for Cultured Cells (Pierce) with a protocol modified by using half the recommended volume of the kit reagents and addition of phosphatase inhibitor cocktail (Sigma), phenylmethylsulfonyl fluoride (PMSF), sodium fluoride, and sodium vanadate, all at 1 mM, in the lysis solution provided in the kit. Thus isolated mitochondria pellets were resuspended for further analysis in 2% 3-[(3-cholamidopropyl)dimethylammonio]-2-hydroxy-1-propanesulfonate (CHAPS) in Tris-buffered saline (25 mM Tris, 0.15 M NaCl; pH 7.2), lysis buffer supplemented with phosphatase inhibiting reagents above, or mitochondrial buffer containing 20 mM HEPES, pH 7.2, 1 mM EGTA, 210 mM mannitol, and 70 mM sucrose, depending on downstream assays.

### MnSOD assay

Cells were treated with H/R as described above in the presence or absence of E2, 1,3,5-tris 4-hydroxyphenyl-4-propyl-1H-pyrazole (PPT), 2,3-bis 4-hydroxyphenyl propionitrile (DPN), 1,3-*Bis*(4-hydroxyphenyl)-4-methyl-5-[4-(2-piperidinylethoxy)phenol]-1*H*-pyrazole dihydrochloride (MPP), or 4-[2-Phenyl-5,7-*bis*(trifluoromethyl)pyrazolo[1,5-*a*]pyrimidin-3-yl]phenol (PHTPP). E2, PPT, and DPN final concentration was 10 nM, and MPP and PHTPP 100 nM. Mitochondrial pellets were isolated as described above and resuspended in mitochondrial cold buffer (20 mM HEPES, pH 7.2, 1 mM EGTA, 210 mM mannitol, and 70 mM sucrose). The mitochondrial MnSOD activity was then measured with a superoxide dismutase kit (Cayman Chemical), according to the manufacturer's protocol, utilizing the dismutation of superoxide radicals generated by xanthine oxidase and hypoxanthine. To ensure the inhibition of possible contaminating extracellular and Cu/Zn SOD activity, potassium cyanide at 1 mM was added to the assays. The resuspended mitochondrial pellets were also immunoblotted for PGM-1 and CoxIV for mitochondrial purity and loading controls, as described below.

### Western/immunoblotting

Western or immunoblotting was performed as reported previously [7]. Briefly, after indicated treatments, cardiomyocytes were washed and lysed. Protein concentration was determined by a bicinchoninic acid-based assay using a commercially available kit (Thermo Fisher Scientific), and 40 µg of protein was loaded onto 10 or 12% SDS-PAGE gel for electrophoresis. Proteins were transferred to polyvinylidene fluoride (PVDF) membrane, which was blocked with 5% milk and probed with primary antibody against the protein of interest, then with anti-rabbit or anti-mouse horseradish peroxidase conjugated secondary antibody (#7074, Cell Signaling Technology) in a standard manner. Primary antibodies used for western blotting include polyclonal antibody against actin (#4968), Cyt *c* (#4272), ATF-2 (#9226), and CoxIV (#4844) from Cell Signaling Technology; SOD-2 (#sc-137254), PGM-1 (#sc-50656), phosphorylated p38 (#sc-17852-R), and p38β (#sc-6187-R) from Santa Cruz Biotechnology. The membrane was then incubated with chemiluminescence reagents using a commercially available ECL Plus Western Blotting Detection System (Amersham Biosciences) and exposed to film. The band intensity on radiographs was determined by densitometry and NIH ImageJ.

### Immunocytochemistry

Rat cardiomyocytes harvested as described above underwent immunocytochemistry to detect intracellular p38β and mitochondria. Cells were plated on glass-bottomed culture dishes coated with poly-d Lysine and incubated with MitoTracker Deep Red 633 (Invitrogen) according to the manufacturer's protocol. Cells were then fixed with ice-cold MeOH at room temperature, washed, and blocked with 10% fetal bovine serum in the dark to suppress non-specific binding. Fixed myocytes were incubated with polyclonal anti-rabbit p38β (#sc-6187-R) and then washed. Next, a fluorochrome-conjugated secondary antibody (Vector Laboratories Inc) was applied in the dark for 45 minutes. Cells were again washed, mounted in anti-fade mounting medium (Vector Laboratories Inc), and examined under a fluorescent microscope.

### Co-immunoprecipitation (Co-IP)

Neonatal rat cardiomyocytes were synchronized overnight in serum-free medium, and H/R was applied as described above. Cells were then lysed in lysis buffer (50 mM Tris HCl pH 7.5, 100 mM NaCl, 5 mM EDTA, 1% Triton X-100, 40 mM β-GP, 200 µM Na_3_VO_4_, 40 mM p-NPP, 1 mM PMSF, protease inhibitor complex, and phosphatase inhibitor complex [Sigma-Aldrich]). p38β or MnSOD from the lysate was then immunoprecipitated overnight at 4°C using polyclonal anti-p38β (Santa Cruz Biotechnology, sc-6187-R) or anti-MnSOD antibody (Santa Cruz Biotechnology, sc-30080), respectively, conjugated to Sepharose beads. The immunoprecipitated protein complex was loaded onto 12% SDS-PAGE gel, electrophoresed and processed for immunoblotting. To determine a molecular interaction between p38β and MnSOD, the p38β-immunoprecipitating complex was blotted for MnSOD and vice versa.

### p38β kinase assay

In-vitro kinase assays were performed with either GST-tagged recombinant human p38β kinase (Sigma-Aldrich) or endogenous p38β immunoprecipitated from rat cardiomyocytes, and MnSOD (Sigma-Aldrich) or recombinant ATF2 fusion protein (Cell Signaling) as substrate, similar to previously described protocols [6], [7]. Briefly, 0.1 µg of active, GST-tagged purified human p38β with specific activity of 123 nmole/min/mg or endogenous p38β immunoprecipitated from rat cardiomyocytes was incubated in kinase reaction buffer (25 mM HEPES, 10 mM magnesium acetate, 40 µM ATP, and 2 mM DTT) at 30°C for 30 min using [γ-32P] ATP and varying concentrations of GST-tagged human recombinant MnSOD or ATF2 as a substrate in the presence or absence of the kinase inhibitor, SB203580 (Sigma-Aldrich) in 1 µM. Thus radiolabeled, phosphorylated substrates were then separated on 12% SDS-PAGE gel and detected by autoradiography, and band density determined by densitometer. Western blotting for immunoprecipitated p38β was performed to demonstrate equal loading of immunoprecipitated kinase used.

### Cell viability assay

After indicated treatments, cultured rat cardiomyocytes were trypsinized, resuspended in PBS, stained with 0.4% trypan blue dye and counted on a hemocytometer. Cells that excluded the dye were considered viable, while those stained blue were nonviable, as described previously [6]. More than three hundred cells were counted per experimental condition in triplicate, and data were analyzed for statistical significance.

### Cytochrome oxidase complex IV activity

Cytochrome oxidase complex IV assay was performed using a commercially available cytochrome oxidase activity colorimetric assay kit according to a manufacturer's instruction (BioVision) after primary neonatal rat cardiomyocytes were treated as specified, and mitochondria isolated from them, as described above. The activity was then normalized to citrate synthase activity, which was assayed using a commercially available kit (ScienCell) according to the manufacturer's instruction after mitochondrial isolation.

### Flow cytometry

Flow cytometry experiments were conducted using a Becton Dickson FACSCalibur flow cytometer (BD Biosciences) after incubating cells with nonyl acridine orange (NAO) at 50 nM for 30 minutes for mitochondrial biogenesis experiments or with JC-1 (5′,6,6′-tetrachloro-1,1′,3,3′-tetraethylbenzimidazolylcarbocyanine iodide) at 15 µM for 15 minutes for mitochondrial membrane potential studies, all at 37°C, per manufacturer's protocol (Molecular Probes). Data from triplicate experiments were analyzed using FlowJo software (Tree Star).

### Mitochondrial anion superoxide

Rat cardiomyocytes were harvested and treated with H/R as above in the presence or absence of E2 at the final concentration of 0.1 nM, 10 nM, or 1 µM. Cells were then stained with MitoSOX™ per manufacturer's instruction. Briefly, after cells were washed with warm HBSS, they were incubated with MitoSOX™ at 5 µM and Hoechst 33342 at 1 µM for 10 min at 37°C, washed, and imaged under fluorescent microscope. MitoSOX™ red fluorescence of 100 cells in one visual field was analyzed with Metamorph and NIH ImageJ software.

### Statistical analysis

All experiments were repeated at least three times. Data were analyzed using two-sided Student-*t* for statistical significance at *p* value<0.05, and the mean ± SE expressed in bar graphs.

## Results

### p38β is present in mitochondria of cardiomyocytes

**Figure 1:**
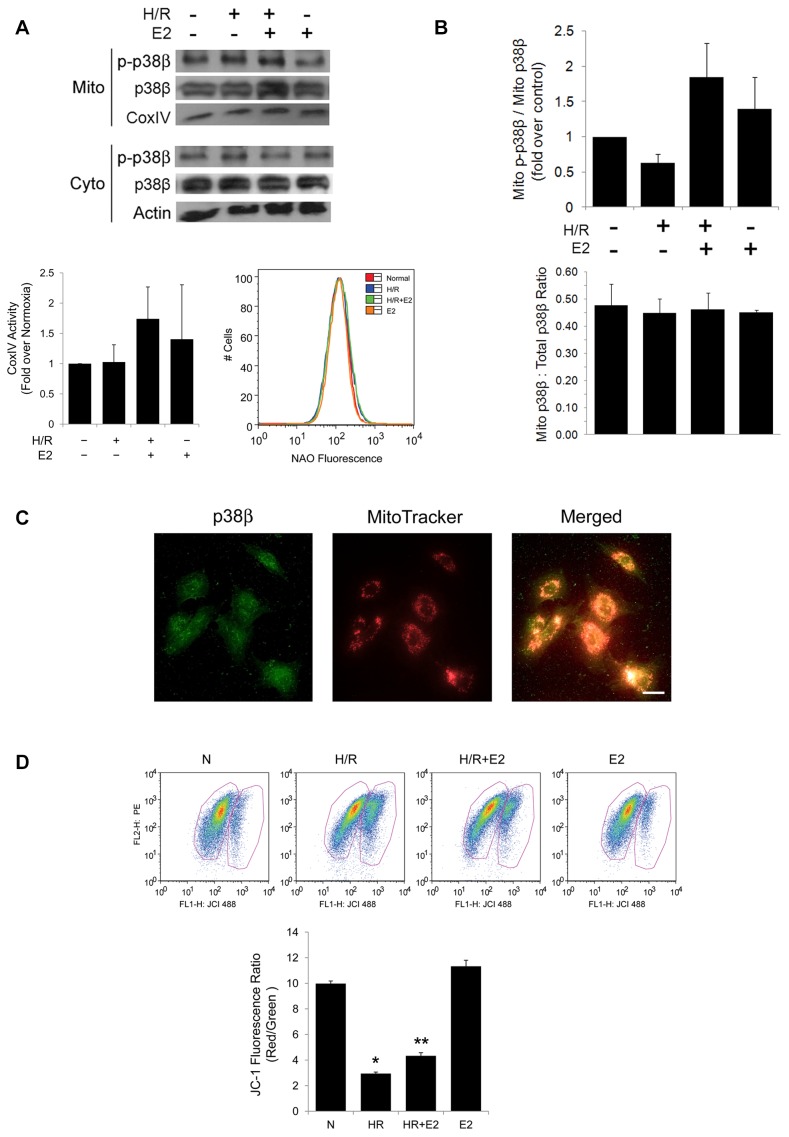
Identification of mitochondrial p38β. *(* ***A*** *)* ***Upper*** **,** Immnoblots of total p38β and phosphorylated p38β (p-p38β) from mitochondria (Mito) and cytosol (Cyto) of rat cardiomyocytes are shown. Final [E2] = 10 nM. Immunoblots of CoxIV and actin are shown also as loading controls for mitochondrial and cytosolic fractions, respectively. ***Lower Left*** **,** CoxIV activity. Mitochondria were isolated and assayed for Cox IV activity and citrate synthase activity. CoxIV activity was then normalized to that of citrate synthase, and is presented with quantitative analysis. ***Lower Right*** **,** NAO fluorescence. Cells were stained with NAO, a marker of mitochondrial biogenesis, and analyzed by flow cytometry. A representative graph from triplicate experiments is presented *(* ***B*** *)* Mitochondrial p-p38β. ***Upper*** **,** the ratio of dual phosphorylated p38β (p-p38β) over total p38β in mitochondria after indicated treatments is expressed in graph. ***Lower*** **,** the percentage of mitochondrial p38β localized to mitochondria is shown in graph as the total mitochondrial p38β over total cellular p38β pool. *(* ***C*** *)* Immunocytochemistry of p38β in cardiomyocytes. Primary antibody specific to p38β is used to stain for intracellular p38β (*left*), and the cells are co-stained with MitoTracker, a fluorescent probe for mitochondria (*middle*). The two images are then merged to demonstrate co-localization of p38β with mitochondria (*right*). The white scale bar represents 25 µm. *(* ***D*** *)* Mitochondrial membrane potential by JC-1 fluorescence. ***Upper*** **,** Flow cytometry analysis of JC-1 stained cells after the indicated treatments shows mitochondrial membrane potential changes reflected by the ratio of the red (FL2 district) and the green (FL1) fluorescent signals. ***Lower*** **,** Red/green JC-1 fluorescence ratio is represented in graph with quantitative analysis. * *p*<0.05 vs. N, ** *p*<0.05 vs. H/R.

Our previous work implicated p38β to be essential in E2-mediated suppression of ROS in cardiomyocytes under stress of H/R. To understand the mechanism behind the antioxidant property of p38β and to determine if p38β is associated with mitochondria, we purified mitochondria from rat cardiomyocytes after H/R with or without a physiological concentration of E2 at 10 nM. The p38β kinase was detected in both cytosolic and mitochondrial fractions by immunoblotting (Fig. 1A, upper). Activation of p38β occurs through phosphorylation at the TGY motif by MAP kinase kinase 6 (MKK6) [8]. The functionally active, phosphorylated p38β kinase (p-p38β) was also present in cardiomyocyte mitochondria. Since there is no antibody specific for p-p38β to our knowledge, we first used antibody specific for dually phosphorylated p38 to immunoprecipitate all phosphorylated p38 isoforms from mitochondria, then blotted the immunoprecipitated complex using antibody specific for the p38β kinase in order to identify p-p38β, similar to published protocols [13]. To demonstrate specificity of the prepared subcellular fractions and as a loading control, the isolated mitochondria from which p38β was immunoprecipitated were blotted for cytochrome *c* oxidase IV (CoxIV), a well-known mitochondrial marker. CoxIV was evenly distributed among the samples. Although there was a nonsignificant trend of increased CoxIV activity (normalized to that of citrate synthase, an exclusive marker of the mitochondrial matrix) with addition of E2, the change was not statistically significant. (Fig. 1A, lower left). Furthermore, there was no significant change in mitochondrial biogenesis among the experimental conditions, determined by flow cytometry of the cells stained with NAO, a marker of mitochondrial mass (Fig. 1A, lower right). These findings are in agreement with the reported neutral effect of acute hypoxia on mitochondrial mass in primary cultured cardiomyocytes [14]. The absence of significant alteration on mitochondrial biogenesis and integrity, reflected by NAO fluorescence, CoxIV protein expression, and its activity support our hypothesis that mitochondrial translocation of p38β is a specific cellular process susceptible to regulation by estrogen, rather than a byproduct of nonspecific mitochondrial disintegration or turnover. To further demonstrate the purity of the preparation, the mitochondrial fractions were blotted for phosphoglucomutase-1 (PGM-1), a cytosolic marker. This protein was absent in our mitochondrial fractions, confirming the purity of the mitochondrial isolation (data not shown). For a loading control of cytosolic fractions, actin was detected by immunoblotting.

The ratio of phosphorylated p38β in mitochondria (mito p-p38β) over total mitochondrial p38β (mito p38β) was increased by E2, indicating that the hormone augments the mitochondrial translocation of the activated kinase (Fig. 1B, upper). Approximately 45% of total cellular p38β localized to mitochondria (Fig. 1B, lower). This percentage, representing the combined pool of unphosphorylated and phosphorylated p38β translocating to mitochondria, was overall not affected by E2, indicating that it is specifically the activated p-p38β subset that E2 promotes into mitochondria.

We also sought to detect mitochondrial p38β by immunocytochemistry (Fig. 1C). Co-staining of p38β and mitochondria was performed on cultured rat cardiomyocytes with p38β-specific antibody and a mitochondria-selective probe (MitoTracker®). The p38β kinase was seen both in nucleus and cytoplasm, particularly in the perinuclear region. This pattern of p38β subcellular distribution has been similarly reported in other cell types [15], [16]. The perinuclear p38β overlapped with fluorescently labeled mitochondria, indicating the presence of the kinase in mitochondria. Combined with the identification of p38β by immunoblotting from the mitochondrial fraction, this demonstrates that the p38β kinase is present in mitochondria of cardiomyocytes. The signal from the fluorescently labeled p38β was relatively low, consistent with our observation and that by others of the moderate level of p38β expression in the heart [17], [18]. To assess functions of mitochondria containing p38β under the experimental conditions, rat cardiomyocytes were stained with JC-1, a fluorescent indicator of the mitochondrial membrane potential (Fig. 1D). Mitochondrial depolarization is indicated by a decrease in the red/green fluorescence intensity ratio, which is dependent only on the membrane potential, and not mitochondrial size, shape or density [19]. As expected, there was a significant alteration in the membrane potential after hypoxia-reoxygenation injury. However, the presence of E2 mitigated the H/R-induced decline in the membrane potential. E2 alone in the absence of H/R did not have a significant effect on the baseline membrane potential.

### Mitochondrial p38β activity

**Figure 2:**
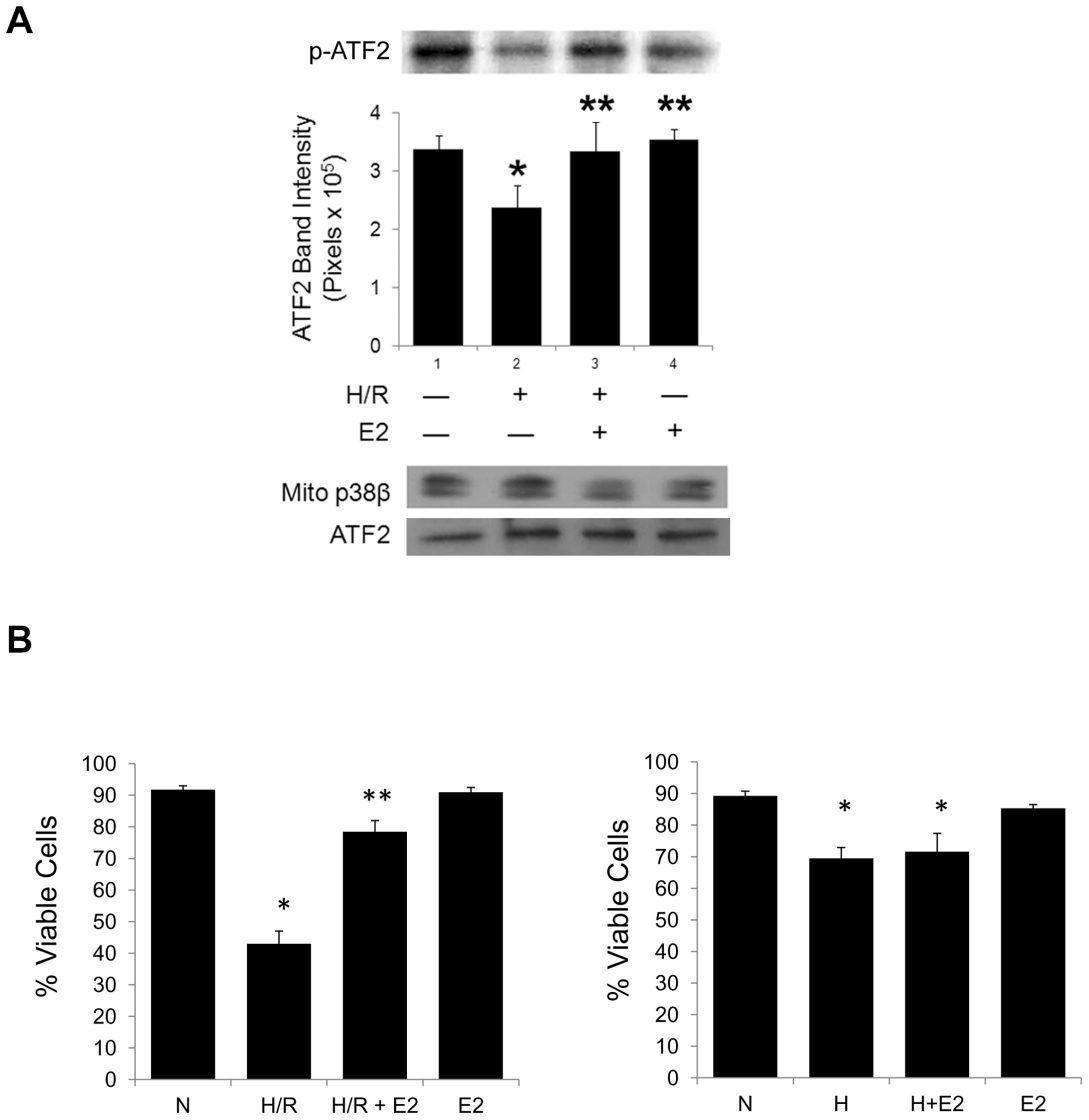
Mitochondrial p38β activity and viability. *(* ***A*** *)* p38β is immunoprecipitated from mitochondria isolated from cultured rat cardiomyocytes and subjected to kinase assays using ^32^P-labeled ATP and ATF2 as its substrate after indicated treatments. Thus radiolabeled ATF2 is shown with quantitative analysis of the band intensity. Representative western blots of ATF2 protein and immunoprecipitated mitochondrial p38β are shown at the bottom as a control. [E2] = 10 nM. * *p*<0.05 *vs.* normoxia or H/R+E2, ** *p*<0.05 *vs.* H/R. *(* ***B*** *)* Viability assay. Cell viability by trypan dye exclusion is shown with the quantitative analysis with H/R (*left*) or hypoxia without reoxygenation (H) (*right*) with quantitative analysis. * *p*<0.05 vs. N, ** *p*<0.05 vs. H/R.

To determine the activity of mitochondrial p38β, we immunoprecipitated native p38β from cardiomyocyte mitochondria and performed in-vitro kinase assays with a well-known substrate, activating transcription factor 2 (ATF2), and [γ-32P] ATP, according to the previously described protocol [6], [8]. Mitochondrial p38β (mito p38β) was functional, evidenced by the radioactive labeling of ATF2 by the kinase (Fig. 2A). Whereas mito p38β activity in H/R stress was lower than that at normoxic baseline, the pre-treatment of cells with E2 prior to H/R yielded a significant recovery of the kinase activity. This is in line with the immunoblotting data (Fig. 1B, upper) demonstrating a higher proportion of activated p38β (p-p38β) in mitochondria after the E2 treatment. As a control, immunoblotting of the kinase protein was also performed, and the activity of the mitochondrial p38β kinase on ATF2 was normalized according to the p38β protein in the reactions. Taken together, we conclude that E2 leads to augmented mitochondrial p38β kinase activation and translocation in cardiomyocytes.

Furthermore, the activation of mitochondrial p38β by E2 correlated positively with E2-driven cardiomyocyte survival benefit (Fig. 2B). Trypan blue dye exclusion assays demonstrated that E2 increased viability of cardiomyocytes after H/R (Fig. 2B, left). However, cell death from hypoxia alone (H) without reoxygenation was not as detrimental as from H/R, with no significant cytoprotection by E2 subsequently, highlighting the protective actions of E2 during reoxygenation (Fig. 2B, right), during which time a majority of oxidative stress is believed to originate and cause cellular damage [20], [21].

### MnSOD activity modulated by E2

Our data demonstrate that there is a mitochondrial pool of functional p38β and that E2 enhances the translocation of the active kinase into mitochondria, a move that correlates with less decline in the mitochondrial membrane potential and better cell survival after H/R stress. We then investigated if the previously reported suppression of oxidative stress by p38β [6] might be attributed to the newly identified mitochondrial pool of the kinase. In cardiomyocytes, mitochondria are the chief source of ROS, which in turn act as a potent inducer of apoptosis if deregulated, such as in H/R [22].

**Figure 3:**
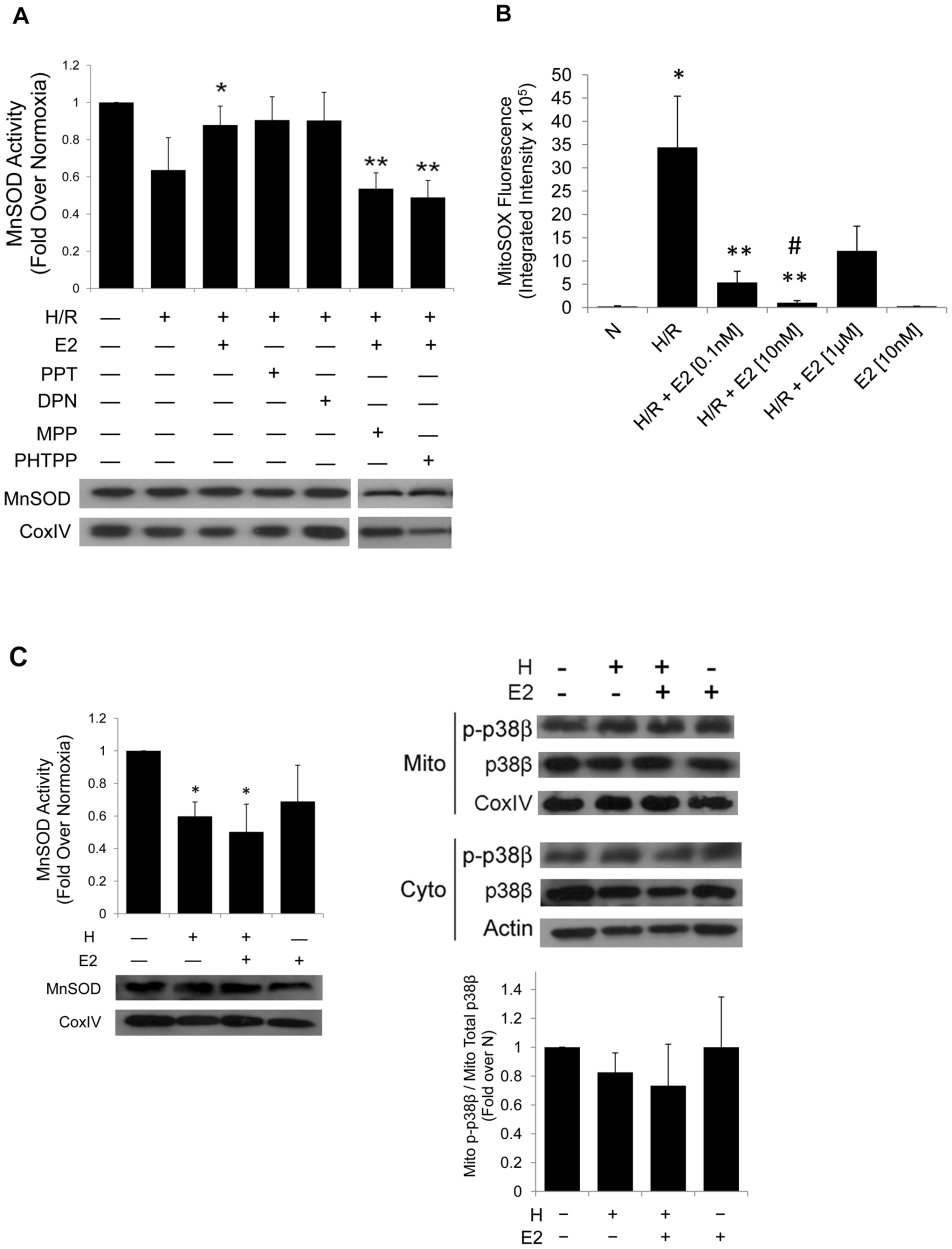
MnSOD activity and anion superoxide. *(* ***A*** *)* After indicated treatments, mitochondria isolated from cardiomyocytes are assayed for MnSOD activity. The final dismutase activity is expressed relative to that under normoxia (control), with quantitative analysis. * *p*<0.05 *vs.* H/R. ** *p*<0.05 *vs.* H/R+E2, H/R+PPT and H/R+DPN. Western blots of the MnSOD protein and CoxIV are shown below for controls. The final concentration of E2, PPT, and DPN was10 nM, and 100 nM for the ER inhibitors, MPP and PHTPP. *(* ***B*** *)* Anion superoxide assay. Intracellular anion superoxide production is determined by MitoSOX™ Red mitochondrial superoxide indicator fluorescence after the indicated treatments and various E2 final concentrations, and presented in graph with quantitative analysis. * *p*<0.05 *vs.* N, ** *p*<0.05 *vs.* H/R, # *p*<0.05 *vs.* H/R, H/R+E2[0.1 nM] or H/R+E2[1 µM]. *(* ***C*** *)* MnSOD activity (*left*) and mito p38β translocation (*right*) after hypoxia without reoxygenation (H) are presented in graph with quantitative analysis. * *p*<0.05 *vs.* N. Western blots of MnSOD, CoxIV protein, and actin are shown for loading control.

MnSOD, also known as SOD-2, is a major part of the mitochondrial antioxidant defense system in the heart. Homozygous mice lacking the MnSOD gene die within 10 days of dilated cardiomyopathy, while heterozygotes exhibit an increased number of apoptotic cardiomyocytes [23], [24]. Considering the notable function of MnSOD in oxidative stress and redox homeostasis, we asked if ROS suppression by E2-mediated p38β previously observed in cardiomyocytes might be via an interaction between the kinase and MnSOD, an important mitochondrial ROS scavenger enzyme. First, we demonstrated the effect of E2 on the MnSOD function by isolating mitochondria from rat cardiomyocytes after H/R stress with or without E2 and assaying for the dismutase activity (Fig. 3A). Potassium cyanide at 1 mM was added to the assay in order to inhibit potential activities from residual cytosolic Cu/Zn SOD and extracellular FeSOD, though MnSOD is the dominant dismutase in mitochondria [25]. With H/R stress, the MnSOD activity was reduced from its baseline at normoxia. By contrast, pre-treating the cells with E2 prior to starting H/R recovered the MnSOD activity to near baseline. Of note, the 30 minute exposure to E2 prior to initiating hypoxia was based on our published observation in which the pre-treatment resulted in the maximal E2-mediated cytoprotection in our cell-based system [7]. The amount of the MnSOD protein from the harvested mitochondria was equal in all samples, as determined by western blotting for the dismutase. This suggests that E2 augments the activity of MnSOD rather than the expressed level of the protein. The amount of mitochondria isolated from lysate was also comparable among the samples, as indicated by the western blot of CoxIV, a mitochondrial marker.

It is also of interest to note that the MnSOD activity was similarly recovered by the activation of each ER subtype separately. This was done through the use of 1,3,5-tris 4-hydroxyphenyl-4-propyl-1H-pyrazole (PPT) or 2,3-bis 4-hydroxyphenyl propionitrile (DPN), agonists for ERα and ERβ, respectively, at 10 nM. On the other hand, use of a specific ER subtype inhibitor, 1,3-*Bis*(4-hydroxyphenyl)-4-methyl-5-[4-(2-piperidinylethoxy)phenol]-1*H*-pyrazole dihydrochloride (MPP), an ERα antagonist, or 4-[2-Phenyl-5,7-*bis*(trifluoromethyl)pyrazolo[1,5-*a*]pyrimidin-3-yl]phenol (PHTPP), an ERβ antagonist, resulted in abrogation of the E2-mediated MnSOD activation. We have previously reported that both ERα and ERβ participate in the E2-triggered suppression of apoptosis following H/R [7]. Our current data demonstrating the involvement of both ER subtypes on the regulation of MnSOD activity provides an additional insight into the mechanism of action by which E2 protects the cardiomyocyte during H/R.

In line with the positive regulation of MnSOD by E2, anion superoxide production after H/R was significantly reduced by E2 (Fig. 3B). Rat cardiomyocytes were stained with MitoSOX™ , a mitochondrial superoxide indicator, after H/R in the presence of varying concentrations of E2. E2 at 0.1 nM and 10 nM significantly decreased superoxide production, while E2 at a higher, non-physiological dose of 1 µM did not. Compared with 0.1 nM, E2 at 10 nM further reduced superoxide production. Thus, E2 concentration of 10 nM (a value within the known physiological concentrations in rats [26]), used in all our previous experiments, yielded the most antioxidant effect.

As mentioned previously, most oxidative stress is generated during the reoxygenation period during H/R. The E2's antioxidant action through p38β is likely to wield the most effect during reoxygenation, as was seen with its protection of cardiomyocytes from cell death (Fig. 2B). We examined, therefore, the influence of E2 on MnSOD activity and mitochondrial p38β during hypoxia without reoxygenation (H). As expected, during hypoxia alone without reoxygenation, the MnSOD activity (Fig. 3C, left) or mitochondrial translocation of activated p38β (Fig. 3C, right) was not supported by E2. Together, they demonstrate that the E2-dependent activation and translocation of p38β, increase in the MnSOD activity, and cell survival benefits are specific to E2 regulation of events during reoxygenation when a majority of oxidative stress is produced.

### MnSOD and p38β interaction

**Figure 4:**
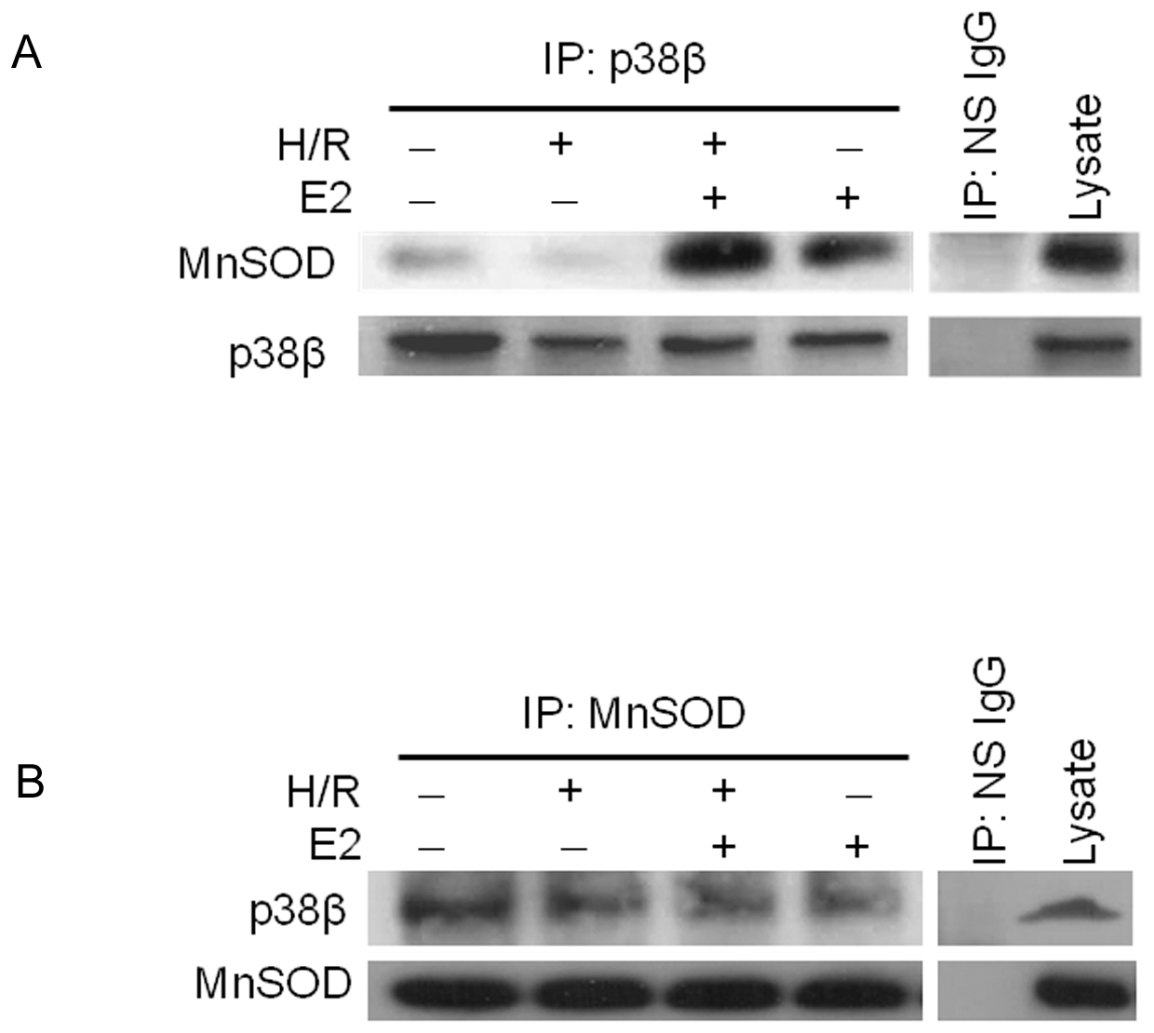
Physical association between p38β and MnSOD. *(* ***A*** *)* Endogenous p38β is immunoprecipitated (IP: p38β) from cardiomyocytes after H/R with or without E2 at 10 nM, and the p38β-containing immunocomplex blotted for MnSOD. A western blot of p38β protein is also shown as a loading control. Nonspecific IgG is used as a specificity control in place of antibody specific to p38β in immunoprecipitation (IP: NS IgG). *(* ***B*** *)* Endogenous MnSOD is immunoprecipitated (IP: MnSOD) from treated cardiomyocytes as indicated, then the MnSOD-containing immunocomplex is blotted for p38β. Nonspecific IgG is used as a specificity control in place of antibody specific to MnSOD in immunoprecipitation (IP: NS IgG).

To see if E2 mediates MnSOD activity via p38β, we first examined if p38β and MnSOD directly interact with each other. After H/R in the presence or absence of E2, p38β was immunoprecipitated from rat cardiomyocytes and the immunocomplexes were blotted for MnSOD (Fig. 4A). At normoxia, there was little MnSOD associated with the p38β immunoprecipitation (IP), indicating a minimal physical interaction between p38β and MnSOD at baseline. This was further attenuated by stress of H/R. With the addition of E2, however, significantly more MnSOD was co-immunoprecipitated with the p38β IP complex, indicating a robust physical association between the kinase and the dismutase induced by E2 (Fig. 4A). When unrelated nonspecific antibody (NS IgG) was used in IP instead of anti-p38β antibody, no MnSOD was identified, attesting to the specificity of the p38β – MnSOD interaction. The IP complexes were also blotted for p38β to demonstrate comparable levels of the kinase immunoprecipitated in each condition. Of note, we have shown previously that p38β activity during H/R is kept low due to the negative control by hypoxia-induced p53, unless E2 is present [7]. The current data presented here demonstrate that the physical interaction of p38β with MnSOD is also reinforced by E2, underscoring the dominantly positive effect E2 exerts on the functionality of p38β.

Conversely, MnSOD was used to co-immunoprecipitate p38β (Fig. 4B). As expected, p38β was identified in the MnSOD immunocomplex, again indicating the physical interaction between the two proteins. As with co-IP above, nonspecific IgG did not pull down p38β in the absence of MnSOD. MnSOD IP was uniform, as shown by blotting of the MnSOD protein. Pulldown of p38β through MnSOD IP was not markedly increased by E2, in contrast to co-IP of MnSOD through p38β. We believe this has to do in part with identifying a protein that is already expressed at a relatively low level, i.e.p38β, indirectly through co-IP with another protein. It also may be due in part to p38β being the primary modulator of MnSOD, and not vice versa, in response to E2. In addition, in our experience, MnSOD IP with the specific MnSOD antibody used (though chosen for its species compatibility and epitope against full length protein) has been relatively inefficient, and may not have fully detected transient E2-mediated interaction between immunoprecipitated MnSOD and native p38β.

Based on these co-IP data, we conclude that p38β and MnSOD physically associate with each other in cardiomyocytes and that this interaction is augmented by E2.

### p38β kinase phosphorylates MnSOD

**Figure 5:**
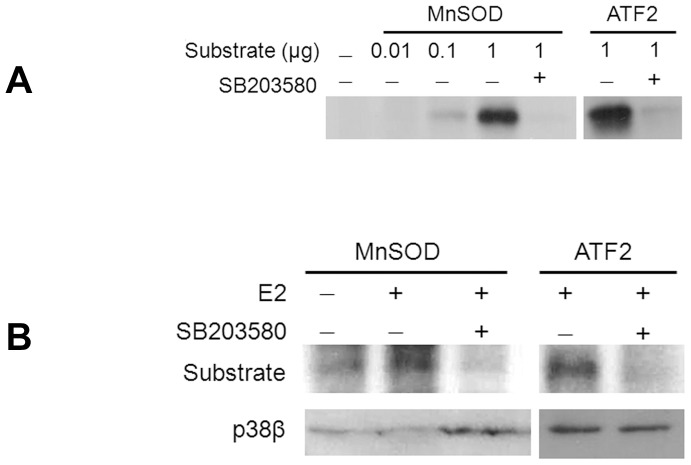
Phosphorylation of MnSOD by p38β kinase. *(* ***A*** *)* In-vitro kinase assay is performed with purified MnSOD in the indicated amount as a substrate in the presence of activated, GST-tagged purified p38β kinase in the presence or absence of the specific kinase inhibitor, SB203580. As a positive control, the kinase activity is also assayed on a well known substrate, ATF2. *(* ***B*** *)* In-vitro kinase assay is performed with endogenous p38β kinase immunoprecipitated from cultured rat cardiomyocytes and 1 µg of MnSOD or ATF2 as substrate in the presence or absence of the kinase inhibitor. A western blot of the isolated kinase is shown as a control.

Given the evidence of a direct association between p38β and MnSOD, we asked if MnSOD was a substrate for the kinase. This would be an important putative mechanism to consider in the context of the p38β effect on ROS suppression and its mitochondrial location, where MnSOD is a major ROS scavenging enzyme. While the transcriptional regulation of the dismutase is well known, there are limited data on the role of post-translational modification, especially that of phosphorylation, in the modulation of MnSOD function [27]. We first performed a kinase assay with active recombinant GST-tagged purified p38β and MnSOD to investigate a potential kinase-substrate relationship (Fig. 5A). Indeed, MnSOD was phosphorylated by p38β in a dose-dependent manner, and this interaction was terminated with a specific chemical inhibitor of the kinase, SB203580. We then confirmed this kinase-substrate relationship by demonstrating MnSOD phosphorylation by the E2-activated endogenous p38β kinase from rat cardiomyocytes (Fig. 5B). Again, MnSOD was phosphorylated by the p38β kinase, and the addition of SB203580 abrogated the reaction, indicating that the phosphorylation of the dismutase was p38β-dependent. For a loading control, the immunoprecipitated p38β MAPK protein in the complex was assessed by western blotting.

In summary, we demonstrate for the first time to our knowledge that there exists a functionally active mitochondrial pool of p38β, and that this kinase phosphorylates MnSOD, a critical component of the mitochondrial antioxidant system, in an E2-dependent manner. The data provide a novel mechanism by which estrogen reduces oxidative stress and protects cardiomyocytes.

## Discussion

Controlled experimental models have collectively shown beneficial actions of E2 on the heart (reviewed in [28]). However, exact molecular mechanisms behind the E2-mediated protection in ischemia-related cardiac injury remain incompletely understood. Evidence supports estrogen's regulation of nitric oxide (NO) and various forms of NO synthase (NOS), particularly endothelial NOS (eNOS) activation [29], [30] via Akt [4], [31]. In cardiomyocytes, p38β is an important downstream target of this E2 signaling pathway. We have previously shown that in cultured rat cardiomyocytes, E2 prevents cell death by downregulating p53 phosphorylation by p38α MAPK and overriding p53-driven inhibition of p38β. Thus activated p38β then suppresses mitochondrial ROS generation, thereby removing a potent trigger for myocyte apoptosis [6], [7].

To date, how E2-mediated p38β effects ROS suppression has not been clear. We now report novel findings that (1) there is a functionally active mitochondrial pool of p38β, (2) the MnSOD activity during H/R is augmented by E2, which attenuates the H/R-induced mitochondrial membrane potential decline and anion superoxide production in cardiomyocytes, and (3) E2-activated p38β interacts with and phosphorylates MnSOD. To our knowledge, this is the first line of evidence to demonstrate that MnSOD serves as a substrate to p38β. Putting together with our previous work identifying p38β as an important mediator of estrogen's antiapoptotic actions, the data provide a unique working model in which E2 signaling to p38β reduces oxidative stress from H/R and promotes cardiomyocyte survival via mitochondrial MnSOD.

Using an online phosphorylation prediction program (NetPhosK 1.0), S106 alone of the MnSOD protein was identified as the residue likely to be phosphorylated by p38. Whether this applies to p38β is unknown, and this may be an important distinction to note because substrate affinity of p38β and that of the other p38 MAPK isoforms varies significantly. This difference may be significant, considering the divergent functionality among the different members of the p38 MAPK family [8], [32], [33]. Therefore, identification of the MnSOD residue(s) phosphorylated by the p38β kinase will be an important aspect of the future effort to further define the mechanism of E2-mediated cytoprotection.

Post-translational modification, such as nitration, S-glutathionylation, glycation, or phosphorylation, of superoxide dismutase (SOD) has been known to alter the dismutase function in the cellular defense against oxidative stress [27]. While some of these post-translational modifications are well-characterized, little is known about the role of MnSOD phosphorylation in cardiac tissue. Modulation of this mitochondrial SOD, however, is crucial to the regulation of redox homeostasis of the heart for normal cardiac development and cardioprotection during stress [23], [24], [34]. We have provided evidence here that E2 upregulates the activity of MnSOD during hypoxia-reoxygenation in cardiomyocytes. In agreement with this finding, it has been reported that E2 increases MnSOD activity in other cell types [35]. Additionally, our data indicate that E2-dependent upregulation of MnSOD activity is not due to a significant alteration in the SOD2 gene expression, as shown by the relatively constant MnSOD protein level during the given duration of H/R and E2 treatment. This is also consistent with data from other cell types, in which the increased dismutase activity by E2 was not associated with the change in SOD mRNA or protein expression during an acute treatment with E2 for less than 24 hours [36], [37]. It is still plausible, however, that a longer exposure to E2 may lead to change in MnSOD gene expression, as shown in unstressed vascular smooth muscle cells [35].

Furthermore, our current study supports involvement of both ERα and ERβ in the modulation of MnSOD activity. This finding extends our previous observation on the participation of both receptors in cardiomyocyte protection from mitochondria-driven apoptosis [7]. Both subtypes are present in mitochondria, although each receptor exhibits its own, distinct subcellular localization pattern upon E2 stimulation [38], [39]. ERs function as dimers, and are traditionally thought to work in homodimers. Nevertheless, a notion of ER heterodimers (ERα/β) may come into play when considering the accumulative data regarding E2-mediated cytoprotection. While not a dominant unit of ER pools, the heterodimers are functionally active in the nucleus and in the plasma membrane [40], [41]. Interestingly, one study utilizing *in silico* modeling technique and molecular dynamics simulation predicts ERα/β heterodimers to be more stable than ERβ/β homodimers [42]. Thus, there is a high likelihood of ERα/β heterodimers existing in mitochondria and participating in the E2-mediated antioxidant and antiapoptotic actions.

This is a study derived from the use of neonatal cardiomyocytes. However, we believe that the findings are relevant and applicable to adult cells, as expression and function of the proteins of interest in neonatal cells are not grossly different from those in adult cells [34], [43], [44], and the experiments were conducted when cells were quiescent, more analogous to differentiated adult cardiomyocytes. Also, though the study was performed in the cell-based system, we believe that insight provided by the mechanistic approach of this study is valuable in understanding the salient details of the molecular events and complements current knowledge gained from in-vivo models.

In summary, we report novel findings of mitochondrial p38β and its action in cardiomyocyte mitochondria. The study sheds light on the molecular mechanism behind the estrogen-mediated cytoprotection and may provide a future direction in developing innovative therapeutic options.
